# Imipramine Inhibits Osteosarcoma Invasion via Src Inactivation and Caspase‐Dependent Apoptosis

**DOI:** 10.1111/jcmm.70761

**Published:** 2025-09-01

**Authors:** Yu‐Chang Liu, Chi‐Jung Fang, Li‐Cho Hsu, Fei‐Ting Hsu, Ming‐Hsien Hu

**Affiliations:** ^1^ Department of Radiation Oncology Chang Bing Show Chwan Memorial Hospital Changhua Taiwan, ROC; ^2^ Department of Medical Imaging and Radiological Sciences Central Taiwan University of Science and Technology Taichung Taiwan, ROC; ^3^ Department of Orthopaedic Surgery An Nan Hospital, China Medical University Tainan Taiwan, ROC; ^4^ Department of Medicine National Yang‐Ming Chiao‐Tung University Hospital Yilan Taiwan, ROC; ^5^ Department of Biological Science and Technology China Medical University Taichung Taiwan, ROC; ^6^ Department of Life Sciences National Central University Taoyuan Taiwan, ROC; ^7^ Department of Orthopedic Surgery Show‐Chwan Memorial Hospital Changhua Taiwan, ROC; ^8^ Department of Post‐Baccalaureate Medicine College of Medicine, National Chung Hsing University Taichung Taiwan, ROC

**Keywords:** apoptosis, imipramine, invasion, osteosarcoma, Src

## Abstract

Osteosarcoma (OS) is an aggressive malignancy characterised by high metastatic potential and poor prognosis. Imipramine, a tricyclic antidepressant, has shown potential anticancer effects. This study evaluates the cytotoxic, pro‐apoptotic and anti‐invasion effects of imipramine on OS cells in vitro and in vivo, as well as its underlying mechanisms. Imipramine significantly reduced U‐2 OS and MG 63 cell viability in a time‐ and dose‐dependent manner, confirmed through MTT and colony formation assays. It induced apoptosis via caspase‐dependent pathways, as evidenced by increased cleaved caspase‐3, ‐8 and ‐9 levels and reduced expression of anti‐apoptotic proteins such as MCL‐1. Imipramine activated both extrinsic and intrinsic apoptosis pathways in vitro and in vivo, increasing Fas, Fas‐L, BAX and BAK while suppressing anti‐apoptotic factors like BCL‐2 and XIAP. Transwell assays showed dose‐dependent inhibition of cell migration and invasion, supported by suppressed Src phosphorylation and downregulation of EMT markers (Snail‐1 and Slug). In U‐2 OS xenograft‐bearing mice, imipramine significantly inhibited tumour growth in a dose‐dependent manner, with the 30 mg/kg group showing the smallest tumour volume and slowest growth rate (*p* = 0.0098). Tumour weights were significantly reduced without impacting body weight or liver and kidney function markers (AST, ALT, γ‐GT and CREA). Histopathological analyses revealed no significant abnormalities in vital organs. Imipramine exerts potent anti‐OS effects by suppressing Src‐mediated invasion and enhancing caspase‐dependent apoptosis through extrinsic and intrinsic pathways. It inhibits tumour progression without inducing systemic toxicity, demonstrating its potential as a therapeutic candidate for OS.

## 1 Introduction

Patients diagnosed with cancer often experience depression and anxiety. Severe symptoms not only reduce their quality of life but also increase both cancer‐specific mortality and all‐cause mortality [1]. During cancer treatment, there has been a growing emphasis on managing depressive symptoms in patients. Healthcare teams are increasingly prioritising the mental health of cancer patients and are actively implementing various interventions to alleviate these symptoms. Previous studies have demonstrated that reducing depressive symptoms can lead to improved survival outcomes in patients with breast cancer, lung cancer and head and neck cancer [2, 3, 4]. Antidepressants can be classified into several types based on their mechanisms of action, such as monoamine oxidase inhibitors (MAOIs), tricyclic antidepressants (TCAs), selective serotonin reuptake inhibitors (SSRIs) and serotonin‐norepinephrine reuptake inhibitors (SNRIs). These medications can improve depressive symptoms and enhance the quality of life for cancer patients [5, 6, 7].

In recent years, the effects of antidepressants on cancer have become an important focus of clinical and preclinical research. Studies on lung, liver, prostate and gastric cancers have found that the use of antidepressants is associated with improved prognosis [8, 9, 10, 11]. Cell and animal models have demonstrated that antidepressants can inhibit tumour cell growth by interfering with the cell cycle and oncogenic kinase signalling pathways. Additionally, they can activate multiple pathways, including apoptosis and autophagy, ultimately leading to tumour cell death [5, 12, 13]. These findings suggest that antidepressants not only benefit mental health but may also offer a potential role in cancer treatment.

Osteosarcoma, a malignant bone tumour, carries a high risk of local recurrence and distant metastasis. Patients often experience feelings of depression stemming from fears of disease recurrence [14, 15, 16]. Previous studies have demonstrated that antidepressants may exert inhibitory effects on osteosarcoma. For instance, SSRIs such as paroxetine and fluoxetine have been shown to constrain osteosarcoma cells' growth and invasion abilities [17, 18]. Developing antidepressants with anticancer potential may contribute to improving therapeutic outcomes and quality of life for osteosarcoma patients.

Imipramine, a tricyclic antidepressant (TCA), is effective in alleviating depressive symptoms, mood disorder and neuropathic pain in cancer patients. Additionally, imipramine has demonstrated tumour‐suppressive effects by inactivating various oncogenic kinases and transcription factors [19]. Our previous studies have shown that imipramine reduces the activity of AKT, extracellular signal‐regulated kinase (ERK), signal transducer and activator of transcription 3 (STAT3) and nuclear factor‐kappaB (NF‐κB), thereby interfering with the progression of lung, oral and breast cancers [20, 21]. However, it is still unclear whether imipramine inhibits the progression of osteosarcoma. Therefore, the primary objective of this study was to evaluate the inhibitory effects and underlying mechanisms of imipramine in osteosarcoma, both in vitro and in vivo.

## 2 Materials and Methods

### 2.1 OS Cell Culture

U‐2 OS and MG63 human osteosarcoma cells were cultured in McCoy's 5A and RPMI‐1640 media, respectively [22, 23]. Both media were supplemented with 10% fetal bovine serum (FBS) and 1% penicillin–streptomycin (PS). Cells were maintained in a humidified incubator at 37°C with 5% CO_2_ until further evaluation. Culture‐related reagents are listed in Table 1.

**TABLE 1:** Reagents used in this study.

Reagents	Company	Cat no. or product no.
DMEM‐HG	Hyclone Laboratories Inc., Utah, UK	SH30003.02
DMSO	Sigma‐Aldrich, St.Louis, MO, USA	CAS 67–68‐5
Fetal Bovine Sera	Hyclone Laboratories	SH30396.02HI
Penicillin–Streptomycin solution	Hyclone Laboratories	SV30010
Pierce BCA Protein Assay Kits	Thermo Fisher Scientific, Waltham, MA, USA	23,225
Trypsin EDTA Solution	Sartorius, Germany	03–079‐1B
Matrigel	Corning, NY, USA	356,237
Zoletil 50	Virbac, Carros Cedex, France	Zoletil 50
Xylazine (Rompun)	Elanco, Indiana, USA	Rompun

### 2.2 3‐(4,5‐Dimethylthiazol‐2‐Yl)‐2,5‐Diphenyltetrazolium Bromide (MTT) Assay

U‐2 OS and MG63 cells were seeded in 96 wells with 5000 cells and treated with various concentrations of imipramine for 24 and 48 h, respectively. After treatment, 0.5 mg/mL of MTT solution was added to each well, and the plates were incubated for an additional 2 h at 37°C. Cell viability was then assessed using the MTT assay. The medium containing MTT was replaced with 100 μL of DMSO in each well to dissolve the formazan crystals, and absorbance was measured at 570 nm using a Multiskan FC microplate reader (Thermo Fisher Scientific).

### 2.3 Colony Formation

U‐2 OS and MG63 cells were treated with various concentrations of imipramine for 24 and 48 h, respectively. After treatment, a serious increase in cell number was prepared (starting from 10,000) of imipramine untreated and treated cells were seeded into 6 well‐plated and incubated for 14 days. Cell colonies were fixed with a methanol acid (3:1) mixture, stained with 0.5% crystal violet solution, rinsed with double‐distilled water and air‐dried before counting. Colony counting was performed according to the methods described by Chen et al. and Brenner et al. [24, 25].

### 2.4 Flow Cytometry for Apoptosis Analysis

U‐2 OS and MG63 cells were seeded in 6 wells with 10,000 cells and treated with various concentrations of imipramine for 24 and 48 h, respectively. For annexin‐V/PI staining, U‐2 OS and MG63 cells were treated with varying concentrations of imipramine for 48 h. Following treatment, cells were harvested and stained with annexin‐V/PI according to the BD Pharmingen protocol (51‐65,874X and 51‐66211E). To assess extrinsic apoptosis, cells were stained with Fas‐FITC, Fas‐L‐PE and cleaved caspase‐8 according to the manufacturer's protocol (details in Table 2). Additionally, cleaved caspase‐3 was used as a marker for caspase‐dependent apoptosis. After staining, signals from all markers were analysed using a NovoCyte flow cytometer (Agilent Technologies Inc., Santa Clara, CA, USA) and quantified with FlowJo software (version 7.6.1) [26, 27].

**TABLE 2:** Flow cytometry‐related reagents and antibodies were used in this study.

Antibodies	Company	Product no.
Annexin V	BD Pharmingen, San Diego, USA	51‐65,874X
Cleaved‐Caspase‐3	Bio Vision, California, USA	K183‐100‐1
Cleaved‐Caspase‐8	NOVUS Biologicals, USA	NBP2‐54862
Cleaved‐Caspase‐9	Bio Vision	K189‐100‐1
FAS	Invitrogen	53–0951‐82
FAS‐L	BioLegend, San Diego, CA.	306,407
Propidium Iodide	BD Pharmingen	51‐66211E

### 2.5 Transwell Invasion and Migration Assay

U‐2 OS and MG63 cells were seeded at 50,000 cells per 6 cm plate and treated with various concentrations of imipramine for 24 and 48 h, respectively. Matrigel was coated onto transwell inserts 1 day before assessing invasion ability. Then, 10,000 cells were seeded into the upper chamber of a transwell insert to assess migration and invasion patterns following imipramine treatment. After 24 h, non‐migrated or non‐invaded cells were washed off with PBS, and the transwell membrane was then fixed in a 3:1 methanol‐acetic acid mixture for 15 min. The membrane was then stained with 0.1% crystal violet solution for 5 min. Migrated and invaded cells on the transwell membrane were visualised by light microscopy, and the percentage of migration/invasion was analysed using ImageJ software version 1.50 (National Institutes of Health, Bethesda, MD, USA) [28].

### 2.6 Western Blotting

U‐2 OS and MG63 cells were seeded at 50,000 cells per 6 cm plate and treated with various concentrations of imipramine for 24 and 48 h, respectively. Cells were lysed in RIPA buffer containing protease and phosphatase inhibitors for 1 h on ice. The same amount of proteins from each group were separated by 6%–10% SDS‐PAGE and transferred onto PVDF membranes. Blocking and incubation with various primary antibodies (Table 3) were then performed for further analysis. Detailed procedures are provided in previous studies [29].

**TABLE 3:** Antibodies for western blotting.

Primary antibody	Catalogue number	Company	Secondary antibody	WB dilution
Cleaved caspase‐3 (Asp175)	#9661	Cell Signalling Technology, MA, USA	Rabbit	1:1000
MCL‐1	3035–100	Biovision, CA, USA	Rabbit	1:1000
Fas (C18C12)	#4233	Cell Signalling Technology	Rabbit	1:1000
FasL (D1N5E)	#68405	Cell Signalling Technology	Rabbit	1:1000
Cleaved caspase‐8 (Asp391) (18C8)	#9496	Cell Signalling Technology	Rabbit	1:1000
Src (Tyr416)	#6943S	Cell Signalling Technology	Rabbit	1:1000
Src	#2109S	Cell Signalling Technology	Rabbit	1:1000
Slug	#9585S	Cell Signalling Technology	Rabbit	1:1000
Snail‐1	#3879S	Cell Signalling Technology	Rabbit	1:1000
Vinculin	PA5‐29688	Invitrogen	Rabbit	1:2000

### 2.7 U‐2 OS Bearing Mice

Six‐week‐old male NOD/SCID mice were purchased from National Laboratory Animal Center (Taipei, Taiwan) and the experiment was approved by China Medical University Animal Ethics Committee (Permit Number: CMU‐SN‐IACUC‐SN2024‐090). U‐2 OS cells with 5 million amounts were inoculated into mice (NOD/SCID mice) right flank for 10 days. A total of fifteen mice were randomly separated into three groups: 0 mg/kg imipramine (0.1% DMSO gavage), 15 mg/kg imipramine (gavage) and 30 mg/kg imipramine (gavage). Tumour size was measured with callipers every 2 days after treatment initiation and calculated using the formula: volume (mm^3^) = 0.523 × length (mm) × width (mm) × height (mm). Mice were sacrificed on day 11 after treatment [30]. Mice serum was isolated on day 10 for biochemistry analysis, including aspartate aminotransferase (AST), alanine aminotransferase (ALT), gamma‐glutamyltransferase (γGT) and creatine (CREA).

### 2.8 Immunohistochemistry (IHC) Staining

Mice tumour tissues were isolated, fixed by 4% formaldehyde and embedded in paraffin. Bio‐Check Laboratories Ltd. (New Taipei City, Taiwan, ROC) sliced the tissue into 50 μm. The IHC staining was followed by the protocol from the IHC Select HRP/DAB kit (Millipore, Burlington, MA, USA) [31, 32]. Antibodies used in IHC staining were listed in Table 4. Specifically, we performed a semi‐quantitative analysis of IHC‐stained tissue sections using ImageJ (NIH) with the IHC Toolbox plugin. Images were captured under identical exposure conditions, and DAB‐positive staining was separated from the haematoxylin background using colour deconvolution. The percentage of positively stained area (or integrated optical density, where appropriate) was then calculated for each field across three randomly selected samples with 3 high‐power fields per sample.

**TABLE 4:** Antibodies for IHC.

Antibodies	Company	Product no.
Src (Tyr 416)	Cell Signalling Technology, MA, USA	#6943S
Cleaved caspase‐3	Cell Signalling Technology, MA, USA	#9661S
Cleaved caspase‐8	Cell Signalling Technology, MA, USA	#9496S
Cleaved caspase‐9	Invitrogen, MA, USA	PA5‐105271
BCL‐2	Cell Signalling Technology, MA, USA	#15071S
MCL‐1	Biovision, CA, USA	3035–100
c‐FLIP	Cell Signalling Technology, MA, USA	#8510S
XIAP	Invitrogen, MA, USA	PA5‐29253
BAK	Cell Signalling Technology, MA, USA	#12105S
BAX	Proteintech, IL, USA	50,599–2‐Ig
Slug	Cell Signalling Technology, MA, USA	#9585S
Snail‐1	Cell Signalling Technology, MA, USA	#3879S
FAS	Elabscience, TX, USA	E‐AB‐40063
FASL	Elabscience, TX, USA	E‐AB‐31410

### 2.9 Haematoxylin and Eosin (H&E) Staining

Heart, lung, liver, spleen, kidney and small intestine were isolated, fixed by 4% formaldehyde and embedded in paraffin. Bio‐Check Laboratories Ltd. (New Taipei City, Taiwan, ROC) sliced the tissue into 50 μm. H&E staining was followed by the protocol as described in previous works [33, 34].

### 2.10 Statistical Analysis

The results are presented as mean ± standard error mean (SEM) from three separate experiments. Statistical comparisons with the control or imipramine treatment groups were conducted using one‐way ANOVA, followed by Tukey's Multiple Comparison Test. For comparisons between just two groups, the Student *t*‐test was employed, with *p* < 0.05 deemed statistically significant.

## 3 Results

### 3.1 Imipramine Induces Cytotoxicity and Caspase‐Dependent Apoptosis of OS Cells

**FIGURE 1:**
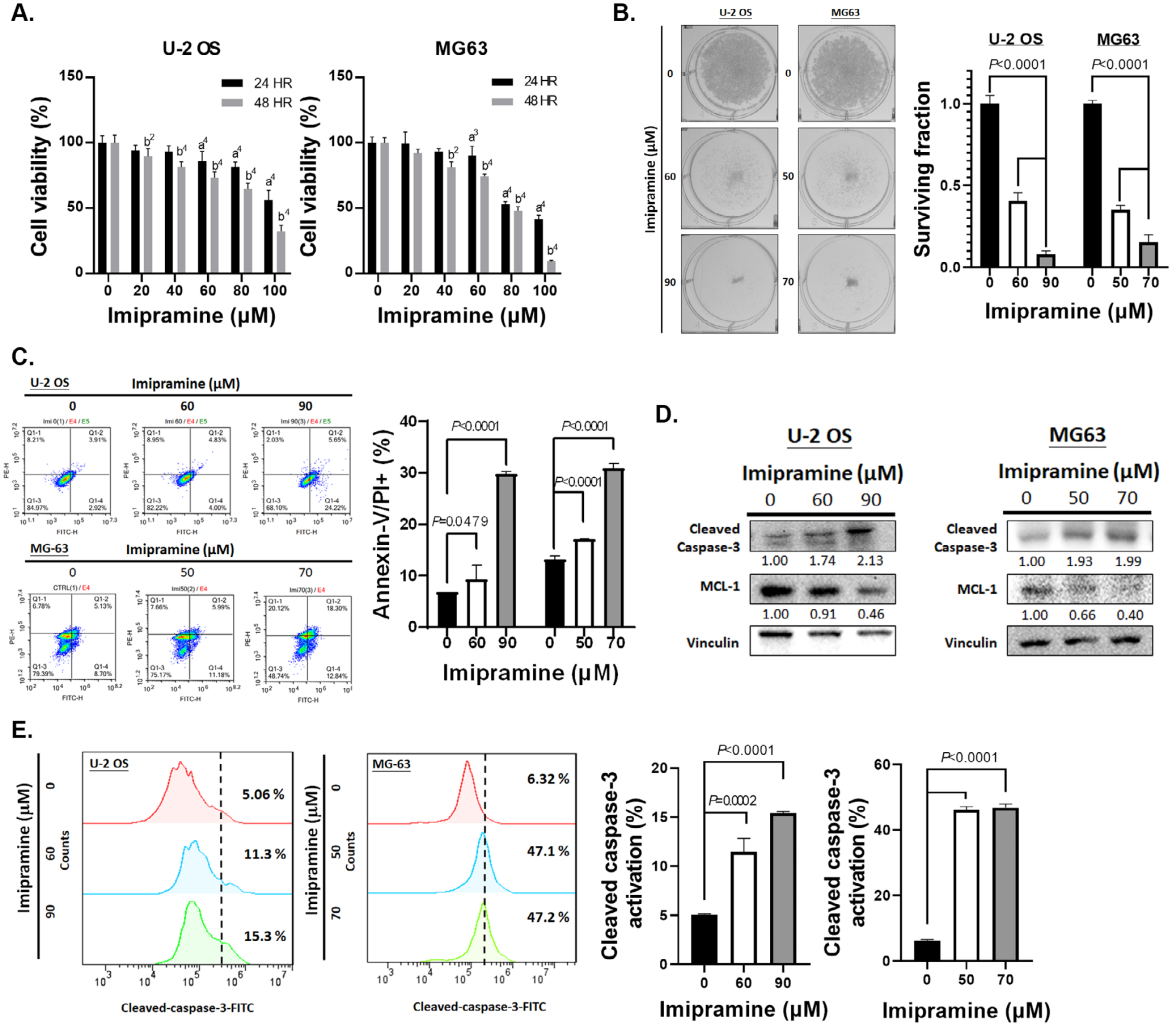
The cytotoxicity and apoptosis effect of imipramine on OS cells. (A) U‐2 OS and MG 63 cells treated with 0–100 μM imipramine for 24 and 48 h and assayed by MTT. (B) Colony formation of U‐2 OS and MG 63 cells after pre‐treated with different concentrations of imipramine for 48 h. (C) The staining of annexin‐V and PI after imipramine treatment for 48 h was assayed by flow cytometry on OS cells. (D) The protein expression of cleaved caspase‐3 and MCL‐1 after imipramine treatment for 48 h was assayed by Western blotting. (E) The activation of cleaved caspase‐3 after imipramine treatment for 48 h was determined by flow cytometry. (a vs. 0 μM 24 h; b vs. 0 μM 48 h; ^2^ *p* < 0.001; ^3^ *p* < 0.0005; ^4^ *p* < 0.0001). MTT assays and flow cytometry analyses were performed in five independent replicates, while Western blotting experiments were conducted in triplicate.

First, we assessed the cytotoxicity of imipramine in U‐2 OS and MG 63 cells using the MTT assay. As shown in Figure 1A, cell viability decreased in both U‐2 OS and MG 63 cells in a time‐ and dose‐dependent manner. Based on the MTT assay results, we selected treatment doses of 0, 60 and 90 μM for U‐2 OS and 0, 50 and 70 μM for MG 63 cells for further evaluation.

Figure 1B illustrates that the surviving fraction from the colony formation assay confirmed the growth inhibition of U‐2 OS and MG 63 cells by imipramine, with significant suppression observed (*p* < 0.0001) compared to the untreated group. Subsequently, we investigated the apoptotic effects of imipramine on these cell lines. As indicated in Figure 1C, imipramine markedly induced positive staining for annexin V and propidium iodide in a dose‐dependent manner in both U‐2 OS and MG 63 cells.

To determine the mechanism of apoptosis, we examined whether it was associated with the caspase‐dependent pathway through Western blotting and flow cytometry. Figure 1D shows that imipramine treatment resulted in increased cleavage of caspase‐3. Conversely, the anti‐apoptotic protein MCL‐1, which inhibits caspase‐dependent apoptosis [35], was also downregulated by imipramine in both U‐2 OS and MG 63 cells. Notably, cleaved caspase‐3 activation was increased 2–3 fold in U‐2 OS cells and 7–8 fold in MG 63 cells following imipramine treatment (Figure 1E).

In conclusion, imipramine induces cytotoxicity and apoptosis in OS cells, which is associated with the caspase‐dependent apoptosis pathway.

### 3.2 Imipramine Induces Caspase‐Dependent Extrinsic and Intrinsic Apoptosis Signalling of OS Cells

**FIGURE 2:**
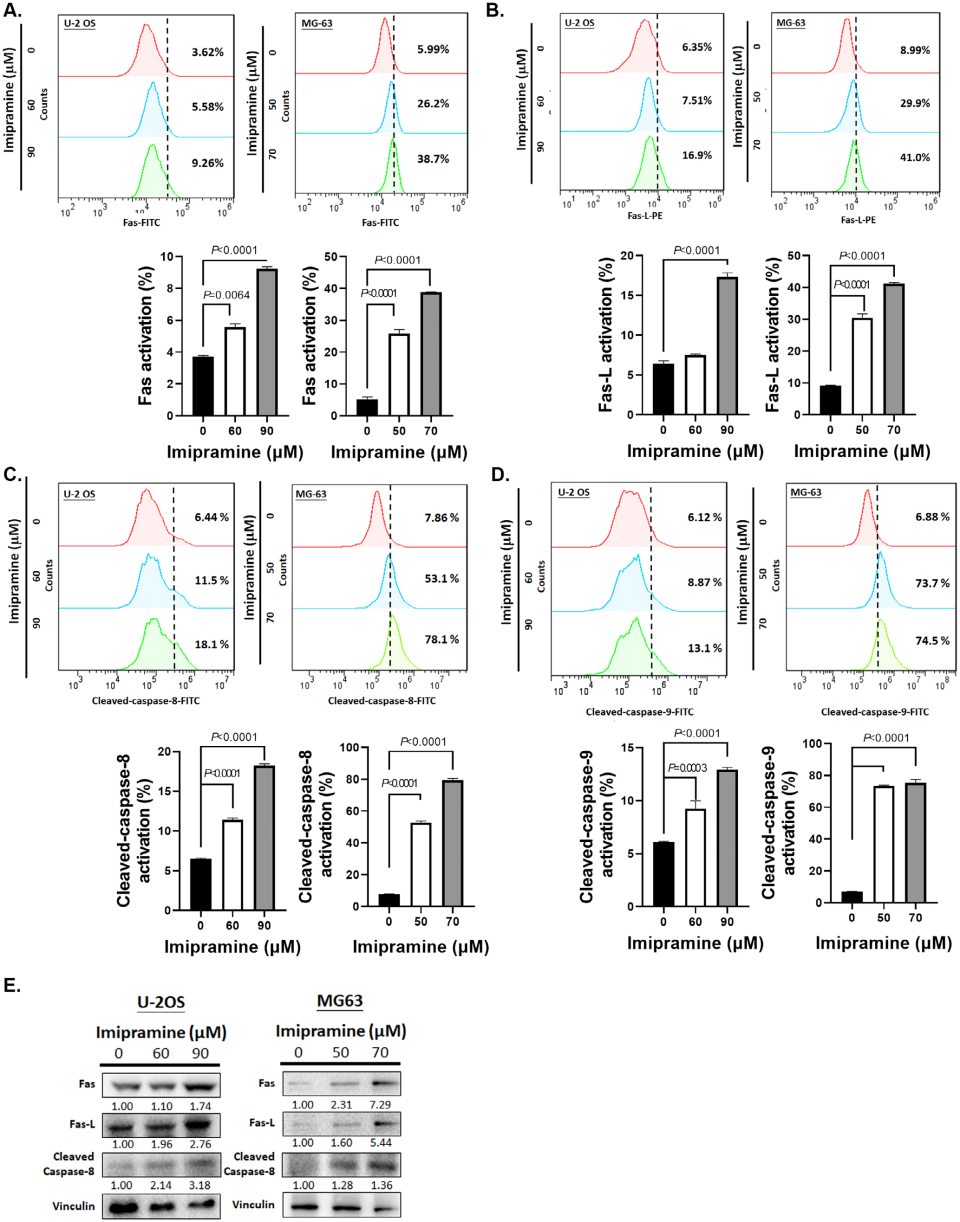
Activation of extrinsic and intrinsic apoptosis signalling pathways by imipramine in OS cells. The expression pattern and quantification bar chart of (A) Fas, (B) Fas‐L, (C) cleaved caspase‐8 and (D) cleaved caspase‐9 after imipramine treatment for 48 h on U‐2 OS and MG63 cells. (E) The protein expression of Fas, Fas‐L and cleaved caspase‐8 after imipramine treatment for 48 h was assayed by Western blotting on U‐2 OS and MG63 cells. Flow cytometry analyses were performed in five independent replicates, while Western blotting experiments were conducted in triplicate.

The apoptotic‐signalling cascade consists of two primary pathways: the extrinsic pathway, which involves the activation of death receptors such as Fas and its ligand (Fas‐L), tumour necrosis factor receptors (TNFRs) and the cleavage of caspase‐8 and caspase‐3, and the intrinsic pathway, which is associated with changes in mitochondrial permeability transition, and the cleavage of caspase‐9 and caspase‐3 [36, 37, 38]. In this study, we evaluated whether imipramine induces both extrinsic and intrinsic apoptosis signalling in U‐2 OS and MG63 cells. As shown in Figure 2A,B, imipramine effectively increased the activation of the death receptor Fas and its ligand Fas‐L in both cell lines. Quantification further confirmed the synergistic activation of these surface death receptors following imipramine treatment. Moreover, the downstream effector caspase‐8, a key mediator in the extrinsic pathway [39], was cleaved after imipramine treatment, reinforcing its role in initiating apoptosis through this pathway (Figure 2C). For intrinsic apoptosis, cleaved caspase‐9 was also detected in imipramine‐treated OS cells (Figure 2D). This finding indicates that imipramine disrupts mitochondrial integrity, leading to the release of apoptogenic factors and subsequent activation of caspase‐9 [40]. Following the activation of these pathways, caspase‐3, the final executioner in the apoptotic cascade, was also cleaved, confirming that imipramine successfully initiates apoptosis in OS cells (as previously shown in Figure 1E). Furthermore, Western blot analysis validated the dose‐dependent increase in Fas, Fas‐L and cleaved caspase‐8 protein expression in the imipramine‐treated group (Figure 2E). Collectively, these findings indicate that imipramine effectively triggers both extrinsic and intrinsic apoptosis signalling in OS cells.

### 3.3 Imipramine Suppresses the Invasion and Migration Effect of OS Cells

**FIGURE 3:**
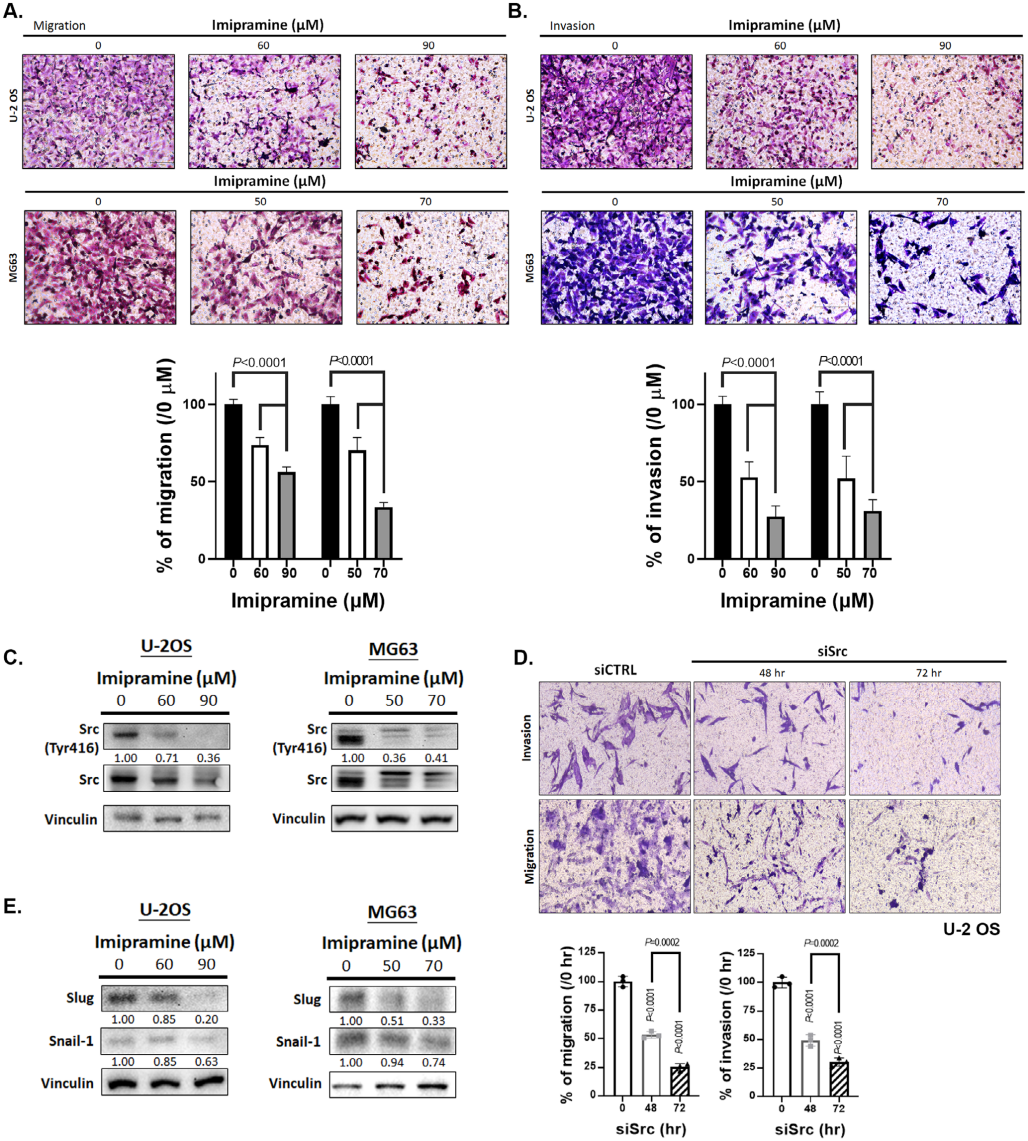
Inhibition of invasion and migration effect of OS cells by imipramine. (A) The migration pattern and quantification bar chart of U‐2 OS and MG63 cells after imipramine treatment for 48 h. (B) The invasion pattern and quantification bar chart of U‐2 OS and MG63 cells after imipramine treatment for 48 h. (C and E) The protein expression of Src (Tyr 416), Snail‐1 and slug after imipramine treatment for 48 h was assayed by Western blotting on U‐2 OS and MG63 cells. (D) The migration and invasion pattern and their quantification bar after silence Src in U‐2 OS cells. Invasion/migration assayed and Western blotting experiments were conducted in triplicate.

To evaluate the potential effects of imipramine on the invasion and migration traits of OS cells, we conducted transwell migration and Matrigel invasion assays as in vitro models of cell motility and invasiveness, along with Western blot analysis of key regulatory proteins. While these assays do not replicate the full in vivo invasion and migration process, they provide established surrogate measures of cellular behaviours associated with invasion and migration potential. In the transwell migration assay, the number of migrated cells was significantly reduced in the 70 and 90 μM imipramine treatment groups for both U‐2 OS and MG63 cells compared to the control group (Figure 3A). Similarly, the invasion ability of U‐2 OS and MG63 cells showed a dose‐dependent decrease with increasing concentrations of imipramine (Figure 3B). To elucidate the underlying molecular mechanisms, we examined the expression of key regulators involved in OS invasion and migration. Western blot analysis revealed that the phosphorylation of Src [41], a known driver of cancer cell migration and invasion, was significantly suppressed by imipramine treatment (Figure 3C). In addition, the suppression of Src by siRNA also illustrated the reduction of invasion and migration effect on OS cells (Figure 3D). Furthermore, key markers of epithelial–mesenchymal transition (EMT), including Snail‐1 and Slug [42], were markedly downregulated in response to imipramine (Figure 3E). These findings suggest that imipramine effectively inhibits OS cell migration and invasion by suppressing Src activation and EMT markers, highlighting its potential as a therapeutic agent targeting invasive and migratory processes in OS.

### 3.4 Imipramine Suppresses the Growth of OS In Vivo

**FIGURE 4:**
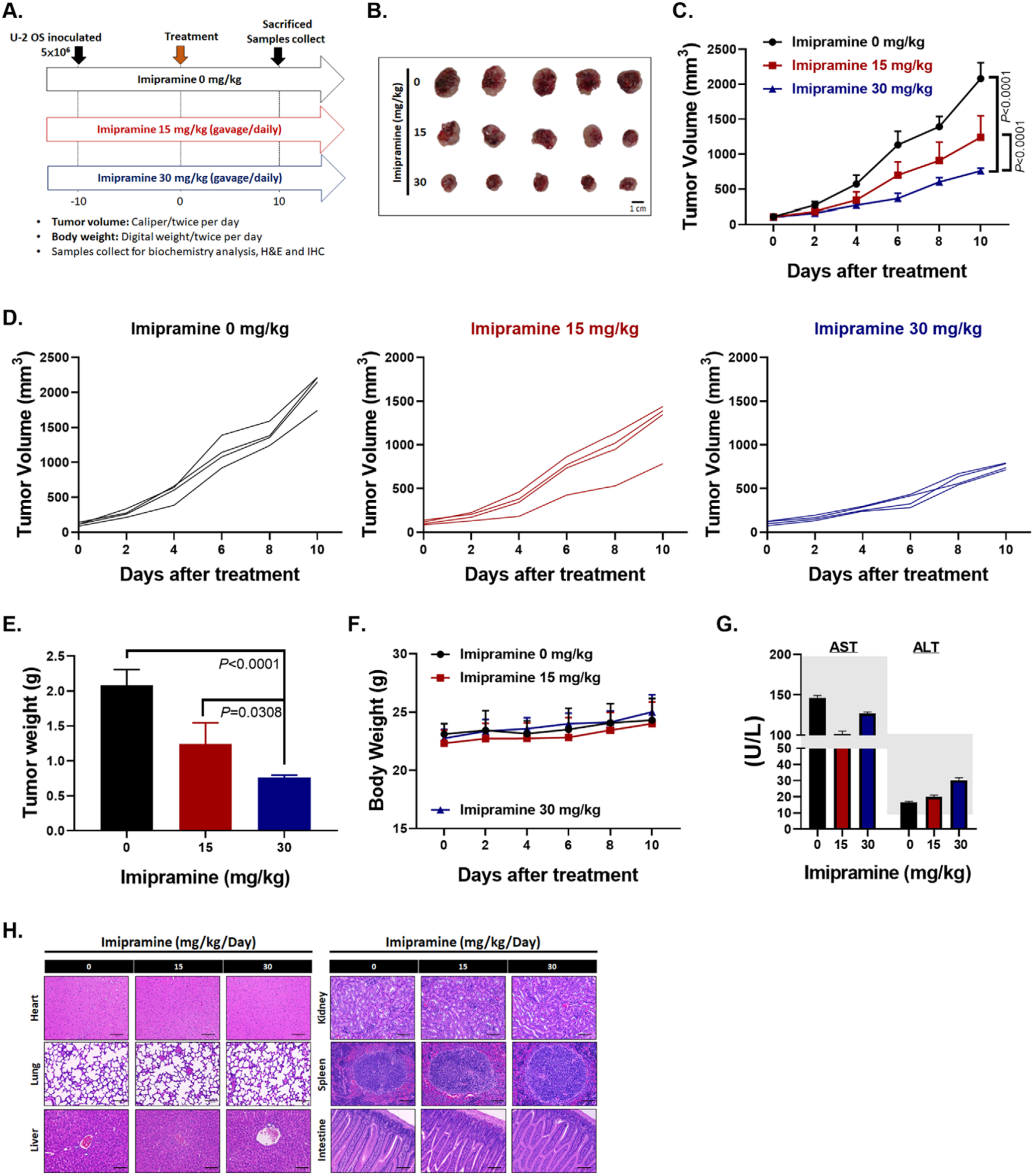
Effects of imipramine on OS tumour growth and systemic health. (A) Experimental flowchart illustrating the imipramine treatment regimen in mice. (B) Images of extracted tumours from mice on day 10 of treatment. (C) Average tumour volume progression across the three groups from day 0 to day 10. (D) Tumour growth trajectories for individual mice in each group. (E) Tumour weight comparison among the three groups on day 10. (F) Body weight trends for the three groups from day 0 to day 10. (G) Serum levels of AST and ALT on day 10 to assess liver function. Gray background represents normal range of these enzymes. (H) H&E staining of heart, lung, liver, kidney, spleen, and intestine tissues to illustrate pathological alteration.

The experimental flow chart of U‐2 OS bearing mice with imipramine treatment (Figure 4A). Tumours were inoculated in mice 10 days before the first treatment. Once the tumours reached a volume of 60 mm^3^, the mice were randomly assigned to three groups: 0, 15 and 30 mg/kg of imipramine, administered daily via gavage. As shown in Figure 4B, the smallest extracted tumours were observed in the group treated with 30 mg/kg of imipramine. Imipramine treatment significantly controlled U‐2 OS tumour progression, with a statistically significant difference between the 0 and 30 mg/kg groups observed after just 4 days of treatment (*p* = 0.0098) (Figure 4C and Table 5). The mean tumour growth time to reach 1000 mm^3^ was 5.09 days for the 0 mg/kg group, 8.84 days for the 15 mg/kg group and 15.23 days for the 30 mg/kg group. This represents a 1.74‐fold slower tumour growth in the 15 mg/kg group and a 3.01‐fold slower growth in the 30 mg/kg group compared to the 0 mg/kg group (Table 6). Individual tumour growth in each group, as depicted in Figure 4D, further demonstrates the suppressive effects of imipramine. Additionally, by day 10, the lightest tumour weight was recorded in the 30 mg/kg imipramine group (Figure 4E). These results highlight imipramine's efficacy in significantly reducing tumour growth and weight in a dose‐dependent manner. We observed that mice body weight remained stable throughout the treatment, indicating the absence of general toxicity (Figure 4F). Furthermore, serum markers of liver function, including AST and ALT (Figure 4G), as well as γ‐GT (Table 7), were maintained within the normal range (grey background) for C57BL/6 mice. This suggests that imipramine treatment did not adversely affect liver function. CREA, an indicator of kidney function, remained unaffected by imipramine treatment (Table 7). Furthermore, no significant pathological differences were observed in the heart, lung, liver, kidney, spleen or intestine among the groups, with only minor score alterations noted in the 0 mg/kg control group (Figure 4H and Table 8). In summary, imipramine significantly inhibited OS progression without impairing normal liver or kidney function.

**TABLE 5:** Statistical analysis of U‐2 OS tumour volume across various doses of imipramine treatment groups and dates.

Tukey's multiple comparisons test	95% CI of difference	Significant	Summary	Adjusted *p*‐value
Day 0				
0 vs. 15 mg/kg	−235.2 to 244.7	No	ns	0.9988
0 vs. 30 mg/kg	−230.3 to 249.6	No	ns	0.9948
15 vs. 30 mg/kg	−235.1 to 244.9	No	ns	0.9987
Day 2				
0 vs. 15 mg/kg	−153.3 to 326.6	No	ns	0.6612
0 vs. 30 mg/kg	−131.0 to 348.9	No	ns	0.5219
15 vs. 30 mg/kg	−217.7 to 262.2	No	ns	0.9728
Day 4				
0 vs. 15 mg/kg	−9.103 to 470.8	No	ns	0.0617
0 vs. 30 mg/kg	63.69 to 543.6	No	**	0.0098
15 vs. 30 mg/kg	−167.2 to 312.8	No	ns	0.7462
Day 6				
0 vs. 15 mg/kg	192.9 to 672.9	No	***	0.0002
0 vs. 30 mg/kg	527.4 to 1007	No	****	< 0.0001
15 vs. 30 mg/kg	94.52 to 574.4	No	**	0.0040
Day 8				
0 vs. 15 mg/kg	244.5 to 724.4	No	****	< 0.0001
0 vs. 30 mg/kg	550.8 to 1031	No	****	< 0.0001
15 vs. 30 mg/kg	66.34 to 546.3	No	**	0.0091
Day 10				
0 vs. 15 mg/kg	599.2 to 1079	No	****	< 0.0001
0 vs. 30 mg/kg	1081 to 1561	No	****	< 0.0001
15 vs. 30 mg/kg	242.1 to 722.0	No	****	< 0.0001

*Note:* **p < 0.01; ***p < 0.005; ****p < 0.001.

Abbreviation: Imip, imipramine.

**TABLE 6:** The mean tumour growth time, delay time and inhibition rate in U‐2 OS tumour‐bearing mice after treatment under different conditions are presented.

Treatment	MTGT (day)^a^	MTGDT (day)^b^	MGIR^c^
Imip 0 mg/kg	5.09	N.A.	N.A.
Imip 15 mg/kg	8.84	3.75	1.74
Imip 30 mg/kg	15.23	10.21	3.01

Abbreviations: Imip, imipramine; N.A., Not available.

^a^ Mean tumour growth time (MTGT): The expected timeframe when the U‐2 OS tumour volume reaches 1000 mm^3^.

^b^ Mean tumour growth delay time (MTGDT): The disparity between the MTGT of the treated group and that of the imipramine 0 mg/kg group.

^c^ Mean growth inhibition rate (MGIR): The MTGT of the treated group divided by the mean tumour growth time of the imipramine 0 mg/kg group.

**TABLE 7:** The serum level of gamma‐glutamyl transpeptidase (γGT, U/L) from U‐2 OS‐bearing xenograft mice is displayed.

Treatment	γGT, U/L	CREA, mg/dL
Imip 0 mg/kg #1	6	0.17
Imip 0 mg/kg #2	7	0.19
Imip 0 mg/kg #3	7	0.20
Imip 15 mg/kg #1	4	0.12
Imip 15 mg/kg #2	4	0.14
Imip 15 mg/kg #3	4	0.16
Imip 30 mg/kg #1	< 3	0.20
Imip 30 mg/kg #2	< 3	0.20
Imip 30 mg/kg #3	< 3	0.20

Abbreviation: Imip, imipramine.

**TABLE 8:** Severity scores for pathological alterations after different doses of imipramine treatment.

Group	Organ type	Score of the region			
		R1	R2	R3	R4
Imip 0 mg/kg#1	Heart	0–1	0–1	0	2
Imip 0 mg/kg#2		0	2	1	0
Imip 15 mg/kg#1	Heart	0	0–1	0	0–1
Imip 15 mg/kg#2		0–1	0	0–1	0–1
Imip 30 mg/kg#1	Heart	0	0–1	0	0
Imip 30 mg/kg#2		0–1	0	0	0
Imip 0 mg/kg#1	Lung	2	0	0	0–1
Imip 0 mg/kg#2		0–1	2	0–1	0
Imip 15 mg/kg#1	Lung	0	0	0–1	1
Imip 15 mg/kg#2		0	0	0	0
Imip 30 mg/kg#1	Lung	0	0	0	0
Imip 30 mg/kg#2		0	0	0–1	0–1
Imip 0 mg/kg#1	Liver	1	2	0	2
Imip 0 mg/kg#2		0–1	1	1	1
Imip 15 mg/kg#1	Liver	1	0–1	0	0–1
Imip 15 mg/kg#2		0	0–1	0	0
Imip 30 mg/kg#1	Liver	0–1	0	0–1	0
Imip 30 mg/kg#2		0–1	0	0–1	0
Imip 0 mg/kg#1	Kidney	0–1	2	2	1
Imip 0 mg/kg#2					
		0	0–1	1	0–1
Imip 15 mg/kg#1	Kidney	0	0	0	0
Imip 15 mg/kg#2		0–1	0–1	0–1	0
Imip 30 mg/kg#1	Kidney	0	0–1	0	0
Imip 30 mg/kg#2		0–1	0	0–1	0
Imip 0 mg/kg#1	Spleen	0–1	2	0	0
Imip 0 mg/kg#2		0	0–1	0–1	0–1
Imip 15 mg/kg#1	Spleen	1	1	1	0
Imip 15 mg/kg#2		0	0	0–1	0–1
Imip 30 mg/kg#1	Spleen	0	0	1	0
Imip 30 mg/kg#2		0–1	0	0	0–1
Imip 0 mg/kg#1	Intestine	0	2	0–1	0–1
Imip 0 mg/kg#2		0–1	1	2	1
Imip 15 mg/kg#1	Intestine	0	0	0–1	0
Imip 15 mg/kg#2		1	0–1	0	1
Imip 30 mg/kg#1	Intestine	0	0–1	0	0
Imip 30 mg/kg#2		0–1	0	1	0–1

*Note:* The alteration of pathology after different treatments was assessed using the following four severity scores: 0—regular tissue, 1—mild changes, 2—moderate changes and 3—significant changes. The pathological findings were interpreted by a veterinarian with over 5 years of experience in pathological interpretation. Each group of mice was examined using two different slices (Slice #1 and Slice #2), with 4 (R1–R4) regions analysed per slice.

Abbreviation: Imip, imipramine.

### 3.5 Imipramine Inhibits OS Progression by Inactivating Src‐Mediated Invasion Signalling and Inducing Caspase‐Dependent Apoptosis

**FIGURE 5:**
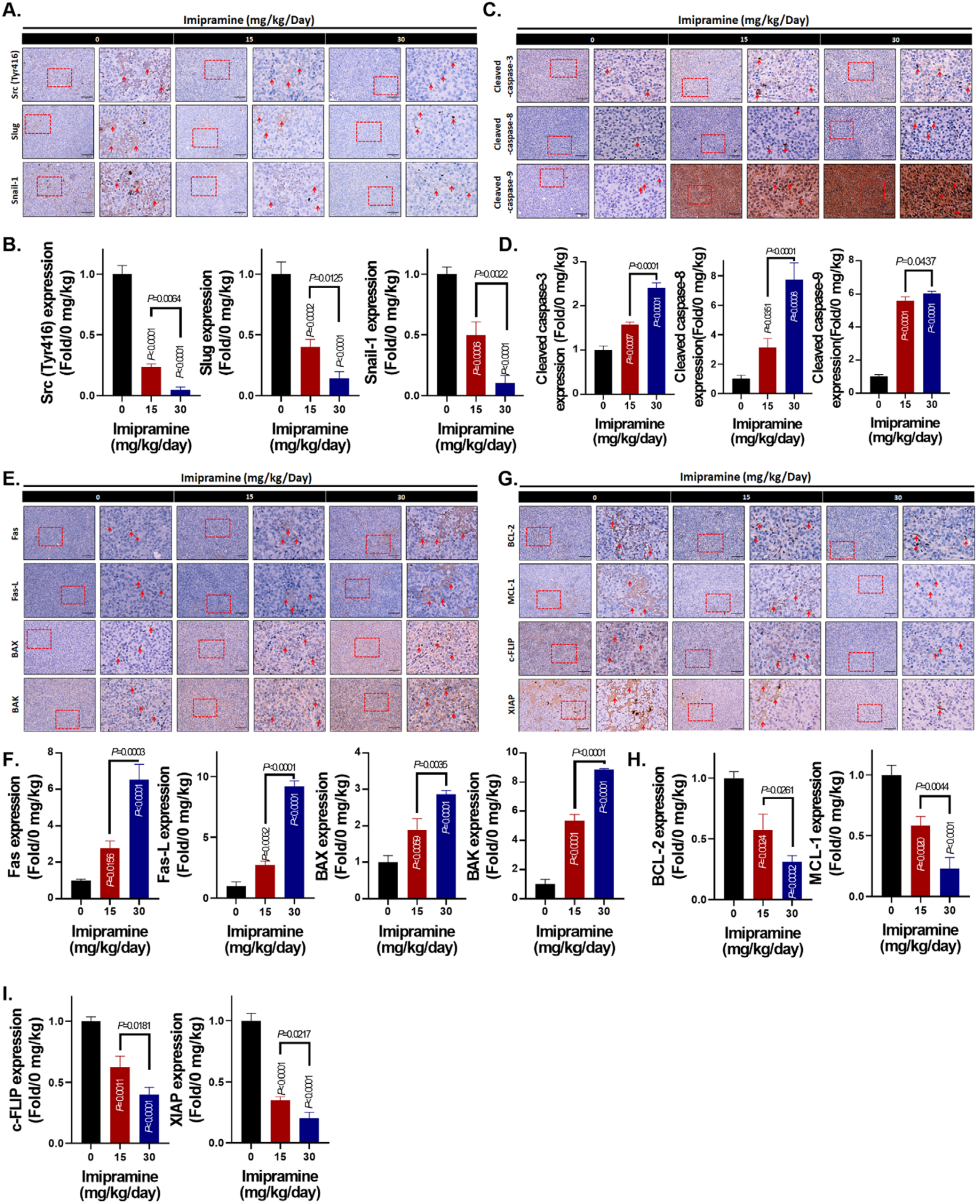
Invasion and migration inhibition and apoptosis induction by imipramine on OS‐bearing model. (A, B) IHC staining pattern and quantification bar chart of Src‐related factors, such as Src (Tyr416), Slug and Snail‐1 in each group. (C, D) IHC staining pattern and quantification bar chart of apoptosis‐related factors, such as cleaved caspase‐3, ‐8 and ‐9 in each group. (E, F) IHC staining pattern and quantification bar chart of extrinsic and intrinsic apoptosis‐related factors, such as Fas, Fas‐L, BAX and BAK in each group. (G–I) IHC staining pattern and quantification bar chart of anti‐apoptosis‐related factors, such as BCL‐2, MCL‐1, c‐FLIP and XIAP in each group. Three representative areas were selected and quantified for each sample in the IHC staining experiment.

**FIGURE 6:**
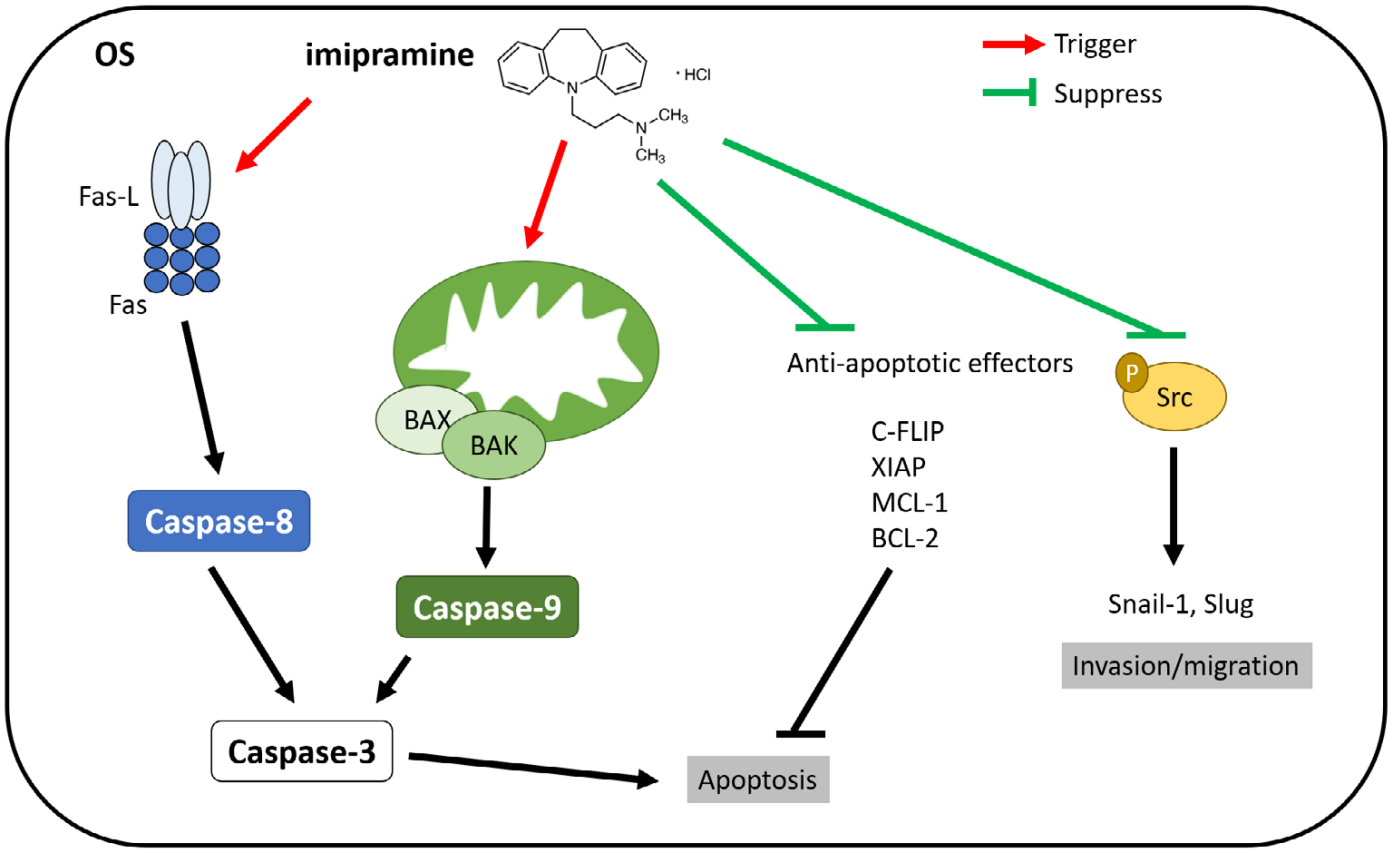
Mechanism of imipramine action in OS cells. Imipramine induces apoptosis in osteosarcoma cells via Fas/Caspase‐8 and mitochondrial BAX/BAK–Caspase‐9 pathways, while suppressing anti‐apoptotic proteins and Src signaling, thereby inhibiting invasion and migration. Red arrows indicate activation, green arrows indicate inhibition.

First, we investigated whether the suppression of phosphorylated Src (p‐Src) and its related factors observed in vitro could also be replicated in tumour tissues in vivo. As shown in Figure 5A, imipramine significantly inhibited p‐Src, Slug and Snail‐1, leading to the inactivation of Src‐mediated invasion signalling pathways [43, 44]. Quantitative analysis demonstrated a suppression of > 50% in p‐Src, Slug and Snail‐1 expression with 15 mg/kg imipramine treatment and 70%–90% with 30 mg/kg treatment (Figure 5B and Table 9A). In addition, caspase family proteins associated with apoptosis, including caspase‐3, caspase‐8 and caspase‐9, were activated by imipramine treatment (Figure 5C). Quantification showed a 2–6‐fold increase in cleaved caspase‐3, ‐8 and ‐9 levels in the imipramine‐treated groups, following a dose‐dependent pattern (Figure 5D and Table 9B). The death receptor family proteins, Fas and Fas‐L, which activate caspase‐8 signalling [45], were also upregulated in the imipramine‐treated groups (Figure 5E). Quantification revealed a 2–10‐fold increase in Fas and Fas‐L levels (Figure 5F and Table 9C). Similarly, mitochondrial apoptosis‐associated proteins such as BAX and BAK, which contribute to caspase‐9 cleavage [46], were induced by imipramine treatment with a 2–8.5‐fold increase (Figure 5G). Conversely, anti‐apoptotic factors involved in both extrinsic and intrinsic apoptosis pathways, including BCL‐2, MCL‐1, c‐FLIP and XIAP [47], were downregulated in response to imipramine treatment, with suppression efficiencies ranging from 50% to 70% (Figure 5H and Table 9D). Taken together, these results indicate that imipramine suppresses Src‐mediated protein expression and anti‐apoptotic protein levels while simultaneously enhancing caspase‐dependent apoptosis factors. This dual mechanism contributes to imipramine's anti‐OS efficacy (Figure 6).

**TABLE 9A:** Statistical analysis of Src mediated metastasis‐related proteins in different doses of imipramine treatment groups on OS model from IHC staining is presented in Figure 5A.

Signalling and EMT‐related proteins				
Tukey's multiple comparisons test	95% CI of difference	Significant	Summary	Adjusted *p*‐value
P‐SRC				
0 vs. 15 mg/kg	0.6496 to 0.8833	Yes	****	< 0.0001
0 vs. 30 mg/kg	0.8369 to 1.0710	Yes	****	< 0.0001
15 vs. 30 mg/kg	0.0705 to 0.3042	Yes	**	0.0064
Snail‐1				
0 vs. 15 mg/kg	0.3020 to 0.7014	Yes	***	0.0006
0 vs. 30 mg/kg	0.6974 to 1.0970	Yes	****	< 0.0001
15 vs. 30 mg/kg	0.1957 to 0.5952	Yes	**	0.0022
Slug				
0 vs. 15 mg/kg	0.4113 to 0.7866	Yes	***	0.0002
0 vs. 30 mg/kg	0.6723 to 1.0480	Yes	****	< 0.0001
15 vs. 30 mg/kg	0.0734 to 0.4486	Yes	*	0.0125

*Note:* *p < 0.05; **p < 0.01; ***p < 0.0005; ****p < 0.0001.

**TABLE 9B:** Statistical analysis of apoptosis‐related proteins in different doses of imipramine treatment groups on OS model from IHC staining is presented in Figure 5C.

Apoptosis related proteins				
Tukey's multiple comparisons test	95% CI of diff.	Significant	Summary	Adjusted *p*‐value
Cleaved caspase‐3				
0 vs. 15 mg/kg	−0.8093 to −0.3446	Yes	***	0.0007
0 vs. 30 mg/kg	−1.6390 to −1.175	Yes	****	< 0.0001
15 vs. 30 mg/kg	−1.0620 to −0.5978	Yes	****	< 0.0001
Cleaved caspase‐8				
0 vs. 15 mg/kg	−4.0430 to −0.1834	Yes	*	0.0351
0 vs. 30 mg/kg	−8.6640 to −4.8040	Yes	****	< 0.0001
15 vs. 30 mg/kg	−6.5510 to −2.6910	Yes	***	0.0008
Cleaved caspase‐9				
0 vs. 15 mg/kg	−5.0200 to −4.1610	Yes	****	< 0.0001
0 vs. 30 mg/kg	−5.4650 to −4.6060	Yes	****	< 0.0001
15 vs. 30 mg/kg	−0.8745 to −0.0155	Yes	*	0.0437

*Note:* *p < 0.05; **p < 0.01; ***p < 0.0005; ****p < 0.0001.

**TABLE 9C:** Statistical analysis of apoptosis‐related proteins in different doses of imipramine treatment groups on OS model from IHC staining is presented in Figure 5E.

Apoptosis‐related proteins				
Tukey's multiple comparisons test	95% CI of diff.	Significant	Summary	Adjusted *p*‐value
Fas				
0 vs. 15 mg/kg	−3.1260 to −0.4357	Yes	*	0.0156
0 vs. 30 mg/kg	−6.8680 to −4.1780	Yes	****	< 0.0001
15 vs. 30 mg/kg	−5.0870 to −2.3970	Yes	***	0.0003
Fas‐L				
0 vs. 15 mg/kg	−2.682 to −0.7944	Yes	**	0.0032
0 vs. 30 mg/kg	−9.123 to −7.235	Yes	****	< 0.0001
15 vs. 30 mg/kg	−7.3850 to −5.497	Yes	****	< 0.0001
BAX				
0 vs. 15 mg/kg	−1.429 to −0.3417	Yes	**	0.0059
0 vs. 30 mg/kg	−2.4110 to −1.3230	Yes	***	0.0001
15 vs. 30 mg/kg	−1.525 to −0.4376	Yes	**	0.0035
BAK				
0 vs. 15 mg/kg	−5.143 to −3.532	Yes	****	< 0.0001
0 vs. 30 mg/kg	−8.643 to −7.033	Yes	****	< 0.0001
15 vs. 30 mg/kg	−4.306 to −2.696	Yes	****	< 0.0001

*p < 0.05; **p < 0.01; ***p < 0.0005; ****p < 0.0001.

**TABLE 9D:** Statistical analysis of anti‐apoptosis‐related proteins in different doses of imipramine treatment groups on OS model from IHC staining is presented in Figure 5G.

Anti‐apoptosis related proteins				
Tukey's multiple comparisons test	95% CI of diff.	Significant	Summary	Adjusted *p*‐value
BCL‐2				
0 vs. 15 mg/kg	0.2082 to 0.6495	Yes	**	0.0024
0 vs. 30 mg/kg	0.4678 to 0.9091	Yes	***	0.0002
15 vs. 30 mg/kg	0.0390 to 0.4803	Yes	*	0.0261
MCL‐1				
0 vs. 15 mg/kg	0.2083 to 0.6208	Yes	**	0.0020
0 vs. 30 mg/kg	0.5640 to 0.9765	Yes	****	< 0.0001
15 vs. 30 mg/kg	0.1495 to 0.5620	Yes	**	0.0044
‐FLIP				
0 vs. 15 mg/kg	0.2128 to 0.5491	Yes	**	0.0011
0 vs. 30 mg/kg	0.4282 to 0.7644	Yes	****	< 0.0001
15 vs. 30 mg/kg	0.0472 to 0.3835	Yes	*	0.0181
XIAP				
0 vs. 15 mg/kg	0.5312 to 0.7692	Yes	****	< 0.0001
0 vs. 30 mg/kg	0.6774 to 0.9155	Yes	****	< 0.0001
15 vs. 30 mg/kg	0.0272 to 0.2653	Yes	*	0.0217

*p < 0.05; **p < 0.01; ***p < 0.0005; ****p < 0.0001.

## 4 Discussion

Combination chemotherapy has been shown to enhance survival rates significantly among osteosarcoma patients. Nevertheless, chemoresistance is one of the significant factors contributing to poor outcomes in these patients. Chemotherapeutic agents, including methotrexate, cisplatin and doxorubicin, are widely employed in osteosarcoma treatment, but their anticancer efficacy may be hindered by anti‐apoptotic proteins such as BCL‐2, MCL‐1, c‐FLIP and XIAP [48, 49, 50, 51, 52]. MCL‐1, a B‐cell lymphoma 2 family member, inhibits apoptosis through the intrinsic pathway by preserving mitochondrial membrane potential [51]. Elevated MCL‐1 expression is associated with osteosarcoma recurrence, whereas its downregulation sensitises osteosarcoma cells to chemotherapy [50, 51]. Our findings demonstrate that imipramine not only reduces MCL‐1 expression in osteosarcoma in vitro, but also significantly inhibits its expression in vivo (Figures 1D and 5G). Moreover, IHC staining demonstrates that the expression of BCL‐2, c‐FLIP and XIAP in tumour tissues is also effectively inhibited by imipramine treatment (Figure 5G,H,I).

In addition to suppressing anti‐apoptotic mechanisms, effectively inducing apoptosis is a critical strategy for ideal anticancer agents to inhibit tumour growth. Apoptosis can be initiated via extrinsic (death receptor) or intrinsic (mitochondrial) pathways, with extrinsic activation primarily involving Caspase‐8 and intrinsic activation primarily involving Caspase‐9. Both pathways ultimately activate effector caspases, including Caspase‐3, ‐6 and ‐7, which mediate apoptosis and lead to tumour cell death [52, 53, 54]. Flow cytometry analysis demonstrates that treatment with imipramine significantly promotes apoptotic events and activates Caspase‐3, ‐8 and ‐9. Furthermore, imipramine induces the expression of cleaved Caspase‐3, ‐8 and ‐9 in osteosarcoma cells (Figures 1E, 2C,D and 5C). The interaction between death receptors and their ligands, such as the Fas/FasL interaction, initiates the extrinsic apoptotic pathway. High Fas expression is associated with a favourable prognosis in osteosarcoma. Moreover, promoting Fas expression has been identified as a key mechanism in reducing osteosarcoma metastasis to the lungs [55, 56]. Additionally, our findings indicate that treatment with imipramine significantly enhances the expression of Fas, as well as the activation of both Fas and FasL (Figure 2A,B,E).

Distant metastasis significantly reduces the 5‐year survival rate of osteosarcoma patients [57]. EMT is a biological process that facilitates the invasion and migration of cancer cells. During EMT, epithelial cells lose their polarity and tight junctions, undergoing a transformation into mesenchymal cells with enhanced invasive properties. Previous studies have shown that osteosarcoma cells exhibit significant characteristics of EMT, accompanied by enhanced migratory and invasive abilities. This process is primarily driven by the upregulation of transcription factors such as Snail and Slug [58, 59, 60]. Fonseca et al. suggested that increased expression of Snail in the cytoplasm is associated with distal metastasis in osteosarcoma patients [61]. The suppression of both Snail and Slug is critical in inhibiting osteosarcoma cell invasion [62, 63]. In this study, we observed that imipramine not only reduces the migration and invasion capabilities of osteosarcoma cells but also downregulates the protein levels of Snail and Slug in both in vitro and in vivo models (Figures 3 and 5A).

The proto‐oncogene c‐Src (Src) is a member of the Src protein tyrosine kinase family, capable of directly or indirectly influencing various oncogenic pathways, including PI3K/AKT/mTOR and Ras/Raf/MEK/ERK. These pathways are involved in promoting tumour phenotypes such as proliferation, anti‐apoptosis and angiogenesis. Additionally, Src can also increase the expression of Snail and Slug, which in turn facilitates the promotion of EMT. Moreover, MCL‐1 expression may be linked to Src activation [43, 64, 65]. Elevated expression levels of Src and its phosphorylated form, p‐Src, have been observed in osteosarcoma and are strongly associated with metastasis and poor patient survival [66]. Src is increasingly recognised as a critical therapeutic target in this context. Clinical trial results indicate that saracatinib, a Src inhibitor, can potentially extend progression‐free survival (PFS) in patients with osteosarcoma. The study reported that patients receiving placebo treatment had a PFS of 8.6 months, while those treated with saracatinib experienced an extended PFS of 19.4 months [67]. The results of our study indicate that imipramine not only reduces p‐Src expression in osteosarcoma cells in vitro, but also significantly diminishes its expression in vivo (Figures 3C,D and 5A).

When selecting an appropriate imipramine dose for animal studies, especially in mouse xenograft models, the dosing must balance efficacy with safety and be guided by prior pharmacological and toxicological data. Preclinical studies have commonly used doses in the range of 10–15 mg/kg (i.p. or oral) for behavioural or antidepressant evaluations, representing the low‐dose range standardly applied in central nervous system models. For anticancer investigations, including tumour growth inhibition in xenograft models, doses between 15 and 30 mg/kg (i.p. or oral, daily) have been shown to achieve biological efficacy with minimal toxicity. Doses exceeding 40–60 mg/kg per day approach the toxicity threshold in mice and are associated with an increased risk of adverse effects such as weight loss and sedation if used over prolonged periods. These dose ranges are supported by prior studies, including J Neurosci Methods [68], Front Oncol [69] and Cancer Lett [70], and our own pilot toxicity assessments confirmed that both 15 and 30 mg/kg were well tolerated over a 14‐day treatment course without significant weight loss or organ toxicity.

Furthermore, recent clinical efforts have begun to explore the antitumour potential of imipramine beyond its conventional use as a tricyclic antidepressant. Although clinical trials specifically targeting osteosarcoma have not yet been initiated, ongoing studies in the United States (NCT03122444, NCT06778434 and NCT04863950) involving patients with skin cancer, breast cancer and brain tumours, as well as a trial in Spain (EudraCT 2021–001328‐17) focusing on colorectal cancer and triple‐negative breast cancer, are currently evaluating the safety and efficacy of imipramine in solid tumours. These investigations reflect a growing interest in repurposing imipramine as a novel anticancer agent. While our study focuses on osteosarcoma, the observed inhibition of Src signalling and induction of caspase‐dependent apoptosis provide mechanistic insights that may support imipramine's broader application across multiple tumour types. Thus, our findings contribute valuable preclinical evidence to guide future translational research and potential therapeutic repositioning of imipramine in oncology.

## 5 Conclusion

In conclusion, our study demonstrates that imipramine effectively induces growth inhibition, apoptosis, suppression of anti‐apoptotic factors and invasion potential in osteosarcoma. Furthermore, treatment with imipramine significantly reduces Src activity and its downstream effector proteins. The induction of apoptosis and inactivation of Src are closely associated with the inhibition of osteosarcoma progression by imipramine. We propose that imipramine holds promising potential as an adjuvant therapy for osteosarcoma, warranting further investigation into its mechanisms and clinical applications.

## 6 Limitation

This study has several limitations. First, we employed U‐2 OS and MG‐63 cell lines xenografted into immunodeficient mice, which do not recapitulate the complexity of the osteosarcoma microenvironment, particularly immune interactions. As a result, the immunomodulatory effects of imipramine—such as potential impacts on macrophages, T cells or immune checkpoint signalling—could not be evaluated. Future studies using immunocompetent or humanised models are needed to clarify these mechanisms.

Second, recent single‐cell RNA sequencing (scRNA‐seq) studies have revealed the remarkable heterogeneity of osteosarcoma (OS), highlighting diverse malignant osteoblast populations and immune cell subsets such as M1/M2 macrophages and exhausted T cells [71, 72, 73]. While our study focused on U‐2 OS and MG‐63 cells to evaluate the direct anti‐tumour effects of imipramine, we acknowledge that these monotypic cell lines do not fully capture the complexity of the OS tumour microenvironment. Furthermore, limited genomic data currently prevent precise matching of these cell lines to the genetic or transcriptional subtypes reported in patient‐derived scRNA‐seq datasets.

## Author Contributions

**Yu‐Chang Liu:** conceptualization (equal), data curation (equal), writing – original draft (lead). **Chi‐Jung Fang:** conceptualization (equal), data curation (equal), writing – original draft (equal). **Li‐Cho Hsu:** conceptualization (equal), data curation (equal), writing – original draft (equal). **Fei‐Ting Hsu:** data curation (supporting), writing – original draft (supporting), writing – review and editing (lead). **Ming‐Hsien Hu:** validation (lead), writing – original draft (lead), writing – review and editing (lead).

## Ethics Statement

The experimental mice were provided by the National Laboratory Animal Center and approved by the Institutional Animal Care and Use Committee (IACUC) at China Medical University (Approval No. CMU‐SN‐IACUC‐SN2024‐090). The animals were housed under a 12‐h light/dark cycle at 25°C in the Animal Housing Facility Center of China Medical University, Taiwan.

## Conflicts of Interest

The authors declare no conflicts of interest.

## Data Availability

The supporting data underlying the findings of this study are available from the corresponding author upon reasonable request.
